# Four Models of HIV Counseling and Testing: Utilization and Test Results in South Africa

**DOI:** 10.1371/journal.pone.0102267

**Published:** 2014-07-11

**Authors:** Tonderai Mabuto, Mary H. Latka, Bulelani Kuwane, Gavin J. Churchyard, Salome Charalambous, Christopher J. Hoffmann

**Affiliations:** 1 The Aurum Institute, Johannesburg, South Africa; 2 School of Public Health, University of the Witwatersrand, Johannesburg, South Africa; 3 London School of Hygiene and Tropical Medicine, London, United Kingdom; 4 Division of Infectious Diseases, The Johns Hopkins University School of Medicine, Baltimore, Maryland, United States of America; Wits Reproductive Health and HIV Institute, South Africa

## Abstract

**Background:**

HIV Counseling and Testing (HCT) is the point-of-entry for pathways of HIV care and prevention. However, HCT is not reaching many who are HIV infected and this may be related to the HCT provision model. We describe HCT utilization and HIV diagnosis using four models of HCT delivery: clinic-based, urban mobile, rural mobile, and stand-alone.

**Methods:**

Using cross-sectional data from routine HCT provided in South Africa, we described client characteristics and HIV test results from information collected during service delivery between January 2009 and June 2012.

**Results:**

118,358 clients received services at clinic-based units, 18,597; stand-alone, 28,937; urban mobile, 38,840; and rural mobile, 31,984. By unit, clients were similar in terms of median age (range 28–31), but differed in sex distribution, employment status, prior testing, and perceived HIV risk. Urban mobile units had the highest proportion of male clients (52%). Rural mobile units reached the highest proportion of clients with no prior HCT (61%) and reporting no perceived HIV risk (64%). Overall, 10,862 clients (9.3%) tested HIV-positive.

**Conclusions:**

Client characteristics varied by HCT model. Importantly, rural and urban mobile units reached more men, first-time testers, and clients who considered themselves to be at low risk for HIV.

## Introduction

HIV Counseling and Testing (HCT) is the first step for multiple interventions for HIV prevention and care. HCT serves as an entry point for HIV prevention services that include prevention of mother-to-child transmission, male medical circumcision, access to condoms, and prevention counseling for HIV-positives [1]–[3]. In addition, linking HIV diagnosed people into care and initiating antiretroviral therapy (ART) is, at an individual level, associated with reduced mortality and extended life expectancy and, at a population level, associated with reduced HIV incidence [4]–[6].

South Africa bears the brunt of one of the largest HIV burdens globally [7]. It is estimated that in 2012, 6.4 million South Africans (12.2%) were living with HIV and that 369,000 new infections occurred among persons aged 15–49 years old [8]. Encouragingly, recent surveys show increases in HCT uptake and awareness of HIV status. In particular, the proportion of persons who reported having ever tested for HIV increased between 2008 and 2012 from 50.8% to 65.5% [8], [9]. Although this marks advancements in HCT scale-up, it is unclear how much these increases reflect an improvement in HCT access for first time testers, hard to reach populations, or underserved communities. Moreover, without frequent testing, a high burden of undiagnosed HIV may still exist despite increased HCT coverage, as shown by a community-based cross-sectional survey in peri-urban South Africa [10].

Ensuring universal and equitable access to HCT services still remains a challenge for many sub-Saharan countries including South Africa [7], [8], [11]. In South Africa HCT utilization is lower among adolescents, the elderly, and men [8]. Low uptake of HCT in South Africa has also been associated with educational level, occupation, fear of involuntary disclosure, and access to HCT [12]–[15]. Furthermore, some groups at higher risk for HIV infection, including men who have sex with men, young women, and possibly other groups may be less likely to receive HCT [16]–[18].

The World Health Organization HCT delivery framework advocates a strategic mix of innovative community-based HCT delivery models and traditional health facility-based HCT to achieve universal and equitable access to HCT [19]. Community-based approaches have been implemented in a variety of settings and with various approaches, aiming at removing structural, logistic, and social barriers to HCT. Such models include fixed stand-alone drop-in HCT, mobile and outreach HCT, workplace and educational center HCT, and door-to-door and index-case home based HCT [19]–[26]. These models may result in earlier HIV diagnosis and may be cost-effective in some settings [20], [22], [23], [27]–[32]. However, data comparing approaches are limited [22], [23], [25], [28], [32]. A better understanding of the nature of clients utilizing different testing strategies may contribute to refining HCT programs. We sought to describe HCT utilization, client characteristics, and associations with test site and with testing HIV-positive in a large standardized program delivering clinic-based, stand-alone, and mobile HCT.

## Methods

### Study design

This research was conducted according to the principles expressed in the Declaration of Helsinki. Routinely collected programmatic data were stripped of all personal identifiers and an analysis dataset was created. The analysis was approved by the University of the Witwatersrand Human Research Ethics Committee. We conducted a cross-sectional study using programmatic data among clients receiving HCT through three delivery models implemented by the Aurum Institute between January 1, 2009 and June 30, 2012. Clients included in this study received HCT services from private practitioner clinic-based, stand-alone HCT, mobile units in an urban/peri-urban area, or mobile units in a rural area, as described below. HCT procedures were standardized across units, provided free of charge, performed by trained counsellors or clinicians, and followed South African National HCT Policy Guidelines on counselling procedures and testing algorithms [33]. All HCT units also provided TB screening using a four-symptom screen. With the exception of clinic-based units, units referred clients for onward care and did not routinely provide additional health services. All three models delivered services within central Johannesburg; two of the models (urban mobile and clinic-based) served an overlapping peri-urban area in Ekurhuleni District; and only one model (rural mobile) was deployed in a rural region of Limpopo Province.

### HIV counseling and testing models

From January 2009 to June 2012, the Aurum Institute provided HCT as part of PEPFAR funded HIV prevention and care programs. Using four service delivery models (Table 1), HCT was provided through coordinated activities with Provincial and District Departments of Health.

**Table 1:** Characteristics of Aurum units providing HIV voluntary counseling and testing between 2009 and 2012.

Model:	Fixed stand-alone HCT	Mobile HCT	Mobile HCT	Fixed clinic-based HCT
Number of sites	1	4	4	20
Setting	Urban	Urban	Rural	Urban & peri-urban
	Johannesburg Central Business District	Johannesburg and Ekurhuleni District	Greater Tubatse, Sekhukhune District	Johannesburg, Tshwane, & Ekurhuleni District
	Major transport hub	Workplaces, community centers, community events, residential areas	Farming areas, workplaces, community centers, residential areas	Private Sector Clinics
Infra-structure	Permanent structure	Minibus with gazebo & two counseling rooms. Minibus returned to office each day.	Minibus with gazebo & two counseling rooms. Minibus returned to office each day.	Permanent structure usually adjacent to a clinic. All sites had at least 1 private room dedicated to HCT
		Placed in industrial areas, or at a workplace in coordination with the enterprise. Also recruited clients from urban areas such as shopping centers.	Visited farms & rural communities adjacent to farms.	
Target population	Commuters, local residents, transport workers	Employed individuals, urban residents	Farm workers, rural residents	Walk-in clients (client initiated HCT)
Client Mobilization	Loudspeakers & pamphlets	Engagement with workplace wellness managers	Engagement with workplace wellness managers	Advertisement in local community
	One-on-one talks & health talks at employee association meetings	Health talks at employee association meetings	Health talks at employee association meetings	HCT staff recruited clients
	Client incentives	Client incentives	Client incentives	
Staff	8 HCT counselors& 3 Professional Nurses	3 HCT counselors &1 Professional Nurse	3 HCT counselors & 1 Professional Nurse	Number varied by clinic
Schedule	Monday – Friday	Monday – Friday	Monday – Friday	Schedule set by clinic
	(08:00–18:00)	(08:00–16:00)	(08:00–16:00)	
	Saturday (08:00–14:00)	Weekends if community events, otherwise closed	Weekends if community events, otherwise closed	The majority were open from Monday to Saturday

Clinic-based: The private practitioner clinic-based HCT was focused on providing client-initiated HCT. These HCT visits were not part of pre-natal care and were mostly client-initiated. Interested individuals were able to register solely for HCT and not have any additional medical evaluation or care. Clients were notified of services through fliers and posters directed toward the immediate catchment area of the clinic. In this study we included data from 20 private practice general practitioner sites; all of them were located in Gauteng Province (central Johannesburg and Ekurhuleni District).

#### Stand-alone

The stand-alone HCT unit was located adjacent to one of the main entrances of a large transport hub in central Johannesburg that served over 100,000 commuters per day, 2,500 resident minibus drivers, and 500 traders [34]. Community engagement workers promoted HCT via public address systems, music, tables with fliers on HIV and other health topics, and placing posters in public spaces that provided schedules and related information regarding HCT availability.

#### Urban and rural mobile

Mobile units were composed of a vehicle equipped with two counseling rooms and a portable gazebo. Prior to testing days, potential clients were notified of the HCT campaign through community messaging or via their employers. On testing days, public address systems and community events directed clients toward services at workplaces, farms, villages, and low-income peri-urban areas. Urban mobile units recruited clients during workplace events, at shopping centers, Department of Health events, and at other settings with anticipated large numbers of potential clients. Rural units traveled to rural communities, farms, public clinics, and community events.

The analysis dataset included socio-demographics, HIV testing history, HIV test results, responses to a four-symptom TB screen (cough, fever, weight-loss, night sweats) and the testing modality. Due to changes in the data collection instruments over the observation period, employment data were only collected from September 2010 to September 2011, perceived HIV risk data from January 2009 to September 2010, and couples testing data from September 2010 to June 2012 (Table 2). We included clients who were at least 15 years old (target population for all HCT sites) and had a recorded HIV-test result. We used the latest HIV result for clients with multiple visits.

**Table 2:** Characteristics of clients testing through Aurum HCT sites from 1 January 2009 to 31 June 2012 (column percentages do not include missing).

Variable	Total	Fixed HCT	Fixed HCT	Mobile HCT	Mobile HCT
	All HCT sites	clinic-based	stand-alone	Urban area	rural area
	(118,358)	(18,597)	(28,937)	(38,840)	(31,984)
	N (%)*	N (%)*	N (%)*	N (%)*	N (%)*
**Age group, years**					
<24	32,490 (30)	4,375 (29)	7,501 (28)	9,781 (26)	10,833 (36)
24–34	38,710 (35)	6,150 (40)	10,426 (40)	13,418 (35)	8,716 (29)
35–45	18,918 (17)	2,543 (17)	4,419 (17)	7,340 (19)	4,616 (15)
>45	19,880(18)	2,165 (14)	3,939 (15)	7,573 (20)	6,203 (20)
Median (IQR)	29 (22–40)	29 (23–38)	29 (23–38)	31 (24–42)	28 (21–41)
Missing†	8,360 (7)	3,364 (18)	2,652 (9)	728 (2)	1,616 (5)
**Sex**					
Men	53,291 (47)	8,367 (50)	12,372 (46)	19,706 (52)	12,846 (41)
Women	59,438 (53)	8,176 (49)	14,651 (54)	18,443 (48)	18,168 (58)
Missing†	5,629 (5)	2,054 (11)	1,914 (7)	691 (2)	970 (3)
**Employment** **					
Unemployed	16,454 (40)	2,649 (45)	2,746 (33)	6,362 (40)	4,697 (45)
Part- time	2,354 (6)	347 (6)	607 (7)	690 (4)	710 (7)
Full-time	14,421 (36)	1,980 (34)	3,277 (40)	6,431 (40)	2,733 (26)
Students	7,203 (18)	881 (15)	1,583 (19)	2,439 (15)	2,300 (22)
Missing†	5,874 (13)	2,454 (30)	1,390 (15)	1,082 (6)	948 (8)
**Prior HIV test**					
Yes	69,790 (59)	11,809 (64)	20,798 (72)	24,626 (63)	12,557 (39)
No	48,350 (41)	6,760 (36)	8,079 (28)	14,122 (36)	19,389 (61)
Missing†	218 (0.2)	28 (0.2)	60 (0.2)	92 (0.2)	38 (0.1)
Prior positive	1,432 (2)	299 (3)	446 (2)	571 (2)	116 (1)
Prior negative	68,358 (98)	11,510 (97)	20,352 (98)	24,055 (98)	12,441 (99)
**Perceived risk** ***					
Yes	14,756 (54)	3,213(56)	4,608 (73)	4,812 (50)	2,123 (36)
No	12,719 (46)	2,505 (44)	1,732 (27)	4,717 (49)	3,765 (64)
Missing†	3,520 (11)	1,243 (18)	1,104 (15)	727 (7)	446 (7)
**Calendar year**					
2009	15,191 (13)	5,150 (27)	3,227 (11)	3,746 (10)	3,068 (10)
2010	22,497 (19)	4,409 (24)	5,706 (20)	8,791 (23)	4,041 (13)
2011	52,862 (45)	7,753 (42)	11,372 (40)	19,191 (49)	14,546 (45)
2012	27,358 (23)	1,285 (7)	8,632 (29)	7,112 (18)	10,329 (32)
**TB type symptoms (any of CFSW)**					
No	113,798 (96)	17,885 (96)	27,477 (95)	37,400 (96)	31,036 (97)
Yes	4,560 (4)	712 (4)	1,460 (5)	1,440 (4)	948 (3)

*Percentage excludes missing data and are based on available data for the variable.

† Category represents proportion of missing data.

**Employment data available from 30/09/2010–30/09/2011.N = 46,306 (49% of total).

***Risk perception data available from 01/01/2009–30/08/2010. N = 30,995 (26% of total).

### Data collection and analysis

We used routinely collected programmatic data. These data were collected on paper forms completed prior to administering an HIV test. All forms were entered into a relational database by trained data capturers. For routine monitoring and evaluation purposes, data were queried for inconsistencies and errors with reconciling using paper forms. We used the Pearson's Chi-square or Kruskal-Wallis tests, as appropriate, to perform comparisons for client characteristics by testing model. Logistic regression was performed to assess associations between a new HIV positive diagnosis and HCT model, perceived HIV risk, sex, and TB symptoms (clients reporting to be previously diagnosed as HIV-positive were excluded from this analysis). Multivariable logistic regression was performed through a step-wise approach which involved fitting a model containing all the study variables followed by sequentially dropping variables (excluding testing site) based on the magnitude of the Wald p-values and biological plausibility. We assessed for two-way interactions between key study variables (age, sex, HCT model, and calendar year) and the dependent variable. We only reported significant interactions. Retention of study variables was based upon Chi-squared p-value of 0.05 or less. Stata version 12 was used for all analysis (College Station, Texas, USA).

## Results

### HCT clients

A total of 121,032 clients received HCT from January 2009 to June 2012. We excluded 2,289 clients because they were less than 15 years old and 385 because HIV test results were missing from the database. Those with missing HIV results were more likely to be women (p = 0.02) and have a prior history of HIV testing (p = 0.003), but were similar in age (p = 0.5), employment (p = 0.1), and perceived HIV risk (p = 0.2) to those included in the analysis. A total of 118,358 HCT client encounters (using the latest encounter for clients with multiple visits) were included from the four HCT models: 18,597 (15.7%) from private practitioner clinic-based, 28,937 (24.5%) from stand-alone, 38,840 (32.8%) from urban mobile, and 31,984 (27.0%) from rural mobile (Table 2). The majority of clients were women (59,438 [53%] of those with recorded sex), the median age was 29 years (interquartile range [IQR] was 22–40), an almost similar proportion of clients reported being unemployed (40%) and full-time employed (36%), the majority reported previous testing for HIV (59%)–2% of whom reported previously testing HIV positive, and 46% reported no perceived risk for HIV infection (Table 2). Of first-time testers, 51% were men and 49% were women.

As a result of changes in data forms and limitations in form completion, data were missing for several variables with the greatest proportion of data missing from the fixed clinic-based units (Table 2). Between 2 and 18% of age data were missing, 2 and 11% of sex data, and 6 and 30% of employment data. Only 0.2% of clients had missing data regarding prior HIV testing.

### Characteristics of HCT clients by HCT model

Although three of the models served a similar geographic region, we found more variation than similarities in important client characteristics by HCT model (Table 2). Similarities were observed between the general practitioner clinic-based and the stand-alone fixed units in testing clients with a similar age distribution (median 29 years, p = 0.06) and reporting a similar proportion of newly diagnosed HIV positive clients (11% and 10%, respectively, p = 0.1). The stand-alone fixed and urban mobile HCT units tested a similar proportion of full-time employed individuals (39.9% and 40.3%, respectively, p = 0.5). The proportion of men that tested was slightly higher in the urban mobile HCT units compared to the clinic based and stand-alone units (52%, 46%, and 50%, respectively, p<0.001).

The proportion with prior HIV testing varied between 63% for the mobile units to 72% for the stand-alone fixed unit (p<0.001), and the proportion with perceived HIV risk ranged from 50% for clients of the mobile units to 73% among those accessing the fixed stand-alone testing (p<0.001). Notably, the proportion of both first-time testers and clients who did not perceive HIV risk varied considerably between the rural mobile HCT and all the urban units, with rural mobile HCT reaching the greatest proportion of first-time HIV testers (61%, p<0.001) and the greatest proportion of clients who did not perceive themselves to be at risk for HIV (64%, p<0.001; Table 2). This high proportion of first time testers and those who did not perceive risk contrasted with only 28% of first time testers and 73% perceiving HIV risk at the stand-alone fixed unit (p<0.001). Among first-time testers, urban mobile HCT units reached the greatest proportion of male testers compared to rural mobile, clinic based and stand-alone units (59.3%, 44.3%, 53.6% and 49.4%, respectively, p<0.001). Both urban and rural mobile units tested a higher proportion of first-time testers who did not perceive themselves to be at risk of HIV compared to fixed clinic based and stand-alone units (63.7%, 51.8%, 47.3% and 25.1%, respectively, p<0.001). Although clinic-based fixed HCT was available in the rural region we do not have the demographic breakdown of clients using those services.

Few clients from any of the settings reported TB type symptoms of cough, fever, night sweats, or weight loss. The proportion ranged from 3% at the rural mobile HCT units to 5% at the stand-alone fixed HCT unit (p<0.001).

### HIV Test Results

A total of 12,294 (9.3%) clients tested HIV positive, of whom 1,432 reported a prior HIV-positive test result (12% of positive results; Table 3). Characteristics associated with a *new* positive result were female sex (odds ratio [OR]: 2.7; 95% confidence interval [CI]: 2.4, 3.1), not having previously tested (OR: 2.3; 95% CI: 2.1, 2.4), being unemployed versus being full-time employed (OR 1.2; 95% CI: 1.1, 1.3), and perceiving risk for HIV (OR: 2.0; 95% CI: 1.9, 2.2; Table 3). Mobile units, especially those providing services in rural areas tested individuals with a lower prevalence of HIV (odds ratio for testing positive at a rural unit was 0.57 (95% CI: 0.54, 0.61) when compared to the urban stand-alone unit). We also observed an interaction between sex and age with a diminishing difference in HIV prevalence between sexes within older age groups.

**Table 3:** Client characteristics associated with a *new* HIV positive diagnosis during HIV counseling and testing.

Variable	% HIV positive	N*	HIV positive	Univariable† odds ratio	Multivariable‡ odds ratio	
			(10,862)	(95% CI)	(95% CI)	
**Sex**						
Male	7.5	52,890	3,986	Referent	Referent	
Female	11	58,451	6,418	1.5 (1.4, 1.6)	2.7 (2.4, 3.1)	
**Age group in years, by sex**						
**Men**						
<24	2.4	12,209	305	Referent	Referent	
24–34	8.2	18,972	1,555	3.6 (3.2, 4. 1)	3.5 (3.0, 3.9)	
35–45	12.6	9,223	1,165	5.8 (5.1, 6.6)	5.8 (5.1, 6.7)	
>45	8.3	9,748	812	3.6 (3.2, 4.2)	3.7 (3.2, 4.2)	
**Women**						
<24	5.3	19,600	1,047	Referent	Referent	
24–34	15.2	18,896	2,876	3.2 (3.0, 3.4)	2.9 (2.7, 3.2)	
35–45	16.6	9,109	1,510	3.5 (3.2, 3.8)	3.2 (2.9, 3.5)	
>45	9.3	9,775	904	1.8 (1.6, 2.0)	1.5 (1.3, 1.6)	
**Employment** **						
Unemployed	12.5	16,166	2,019	1.2 (1.1, 1.3)	1.2 (1.1, 1.3)	
Part- time employed	13.4	2,322	310	1.3 (1.1, 1.4)	1.3 (1.2, 1.5)	
Full-time employed	10.6	14,221	1,507	Referent	Referent	
Students	3.1	7,184	224	0.26 (0.23, 0.31)	0.45 (0.4, 0.5)	
**Previous test**						
No	12.1	48,350	5,843	1.8 (1.7, 1.8)	2.3 (2.1, 2.4)	
Yes	7.3	68,358	4,995	Referent	Referent	
**Perceived risk** **						
No	7.6	12,619	963	Referent	Referent	
Yes	15.6	14,453	2,248	2.2 (2.1, 2.4)	2.0 (1.9, 2.2)	
**HCT site**						
Clinic-based	11.3	18,298	2,064	1.15 (1.1, 1.2)	1.05 (0.98, 1.13)	
Stand-alone	10.0	28,491	2,839	Referent	Referent	
Mobile- urban	9.6	38,269	3,679	0.96 (0.91, 1.01)	0.82 (0.78, 0.87)	
Mobile- rural	7.2	31,868	2,280	0.69 (0.66, 0.74)	0.57 (0.54, 0.61)	

*Excludes missing data.

† Univariable regression was performed using participants with available data for the pairwise comparisons.

‡ Multivariable regression was performed using participants with complete for variables in the model (N = 103273).

**Employment data available from 30 September 2010–30 September 2011.

**Risk perception data available from 01/01/2009–30/08/2010. N = 30,995.

## Discussion

We have found variation in important client characteristics by HCT model. Mobile units, both those deployed in urban and rural areas, reached a larger proportion of people who had not previously been tested for HIV, but also, were less likely to find HIV positives among those tested. In addition, urban mobile units achieved a slightly higher uptake of men, a group that generally has low utilization of traditional clinic-based HCT [12], [20], [27], [32]. Notably, although this study used data from routine programmatic HCT, all the urban models targeted roughly the same urban catchment areas. Thus we believe that the composition of the catchment area did not dictate uptake, but rather the model itself played an important role in uptake.

In the rural areas, we did not provide either stand-alone or facility-based HCT. However, HCT is available at all government primary care clinics, thus we believe that the mobile units improved accessibility and, possibly, acceptability of HCT, leading to a high proportion of first-time testers and clients who did not perceive themselves to be at risk for HIV. We note that the prevalence of HIV among clients at the mobile units was lower than clinic and stand-alone: 7.2% among the rural clients and 9.6% among urban clients. This may reflect the acceptability to a wider range of clients, including lower risk clients who may be less inclined to travel to facilities specifically for HCT. Supporting this hypothesis is that of all the mobile HCT clients in rural areas, 61% had not previously tested for HIV and 64% did not perceive risk factors for having HIV. These findings are similar to those reported from a representative population-based household survey of HIV knowledge in another South African rural setting where 68% of the clients reported no history of HIV testing [13]. The similarity of results in prior testing between our HCT program and a population-based sampling study suggests that the mobile HCT approach may have achieved broad-based uptake and is serving a population who is not accessing other options either due to reasons of access (distance) or acceptability.

Our findings are based on routinely collected programmatic data, which have strengths as well as limitations. They have the strength of a large sample covering three years of HCT that characterizes routine services in South Africa. A limitation is that data collection was not the focus of the program and data are less complete than can be expected from research settings. For example, some variables, such as perceived HIV risk and employment status, were only available for part of the study period as a result of changes in data collection instruments leading to only 26% of participants having perceived risk data and 49% having employment data. We also had missing data that was either not completed or captured in the database. The amount of missing data varied across the HCT sites and participant characteristics, thereby potentially biasing results. While there is no reason to suspect a systematic bias regarding missing data, the proportion of missing data varied by model. We did not impute missing data, and relied on complete case and pairwise deletion methods. Although we delivered urban and peri-urban services to largely overlapping catchment areas, comparison of uptake within one catchment area was not the goal of service implementation. As a result, there may have been differences in the populations served by each model that could potentially contribute to differences in client characteristics.

We believe that our findings, coupled with prior studies, point to the benefits of using multiple modalities to deliver HCT, including the potential value of population-targeted mobile HCT units and stand-alone testing services in high-traffic areas. Notably, where all models were available, these two models (mobile and stand-alone) reached a larger proportion of men and employed individuals – groups that are usually harder to reach. Ideally, several models of HCT should be integrated into a comprehensive HIV service package to achieve the individual and population-level health and prevention potential of early HIV diagnosis.

## References

[1] Allen S, Meinzen-Derr J, Kautzman M, Zulu I, Trask S, et al (2003) Sexual behavior of HIV discordant couples after HIV counseling and testing. AIDS 17: 733–740. 12646797 10.1097/00002030-200303280-00012

[2] Marks G, Crepaz N, Janssen RS (2006) Estimating sexual transmission of HIV from persons aware and unaware that they are infected with the virus in the USA. AIDS 20: 1447–1450. 16791020 10.1097/01.aids.0000233579.79714.8d

[3] Denison JA, O'Reilly KR, Schmid GP, Kennedy CE, Sweat MD (2008) HIV voluntary counseling and testing and behavioral risk reduction in developing countries: a meta-analysis, 1990–2005. AIDS Behav 12: 363–373. 18161018 10.1007/s10461-007-9349-x

[4] Bor J, Herbst AJ, Newell ML, Barnighausen T (2013) Increases in adult life expectancy in rural South Africa: valuing the scale-up of HIV treatment. Science 339: 961–965. 23430655 10.1126/science.1230413 PMC3860268

[5] Tanser F, Barnighausen T, Grapsa E, Zaidi J, Newell ML (2013) High coverage of ART associated with decline in risk of HIV acquisition in rural KwaZulu-Natal, South Africa. Science 339: 966–971. 23430656 10.1126/science.1228160 PMC4255272

[6] Cohen MS, Chen YQ, McCauley M, Gamble T, Hosseinipour MC, et al (2011) Prevention of HIV-1 infection with early antiretroviral therapy. NEnglJ Med 365: 493–505. 10.1056/NEJMoa1105243 PMC3200068 21767103

[7] Joint United Nations Programme on HIV/AIDS (2013) UNAIDS report on the global AIDS epidemic 2013.

[8] Shisana O, Rehle T, Simabyi L, Zuma K, Jooste S, et al. (2014) South African National HIV Prevalence, Incidence and Behaviour Survey,2012. Cape Town. Available: http://www.hsrc.ac.za/en/research-areas/Research_Areas_HAST/HAST_National_HIV_Survey. 10.2989/16085906.2016.1153491 27002359

[9] Shisana O, Rehle T, Simabyi L, Zuma K, Jooste S, et al. (2009) South African National HIV Prevalence, incidence, behaviour and communication Survey 2008: A turning tide among teenagers. Cape Town. Available: http://www.mrc.ac.za/pressreleases/2009/sanat.pdf.

[10] Kranzer K, van SN, Karmue U, Middelkoop K, Sebastian E, et al (2011) High prevalence of self-reported undiagnosed HIV despite high coverage of HIV testing: a cross-sectional population based sero-survey in South Africa. PLoSOne 6: e25244. 10.1371/journal.pone.0025244 PMC3182182 21969875

[11] Joint United Nations Programme on HIV/AIDS (2013) Getting to zero: HIV in Eastern and Southern africa.

[12] Peltzer K, Matseke G, Mzolo T, Majaja M (2009) Determinants of knowledge of HIV status in South Africa: results from a population-based HIV survey. BMC Public Health 9: 174. 19500373 10.1186/1471-2458-9-174 PMC2700104

[13] Tabana H, Doherty T, Swanevelder S, Lombard C, Jackson D, et al (2012) Knowledge of HIV status prior to a community HIV counseling and testing intervention in a rural district of south Africa: results of a community based survey. BMC Infect Dis 12: 73. 22458410 10.1186/1471-2334-12-73 PMC3338079

[14] Venkatesh KK, Madiba P, de BG, Lurie MN, Coates TJ, et al (2011) Who gets tested for HIV in a South African urban township? Implications for test and treat and gender-based prevention interventions. J AcquirImmune DeficSyndr 56: 151–165. 10.1097/QAI.0b013e318202c82c PMC3137901 21084993

[15] Hutchinson PL, Mahlalela X (2006) Utilization of voluntary counseling and testing services in the Eastern Cape, South Africa. AIDS Care 18: 446–455. 16777636 10.1080/09540120500213511

[16] Maughan-Brown B, Nyblade L (2014) Different Dimensions of HIV-Related Stigma May Have Opposite Effects on HIV Testing: Evidence Among Young Men and Women in South Africa. AIDS Behav 18: 958–965. 24101100 10.1007/s10461-013-0636-4

[17] Vu L, Andrinopoulos K, Tun W, Adebajo S (2013) High levels of unprotected anal intercourse and never testing for HIV among men who have sex with men in Nigeria: evidence from a cross-sectional survey for the need for innovative approaches to HIV prevention. Sex Transm Infect 89: 659–665. 23851190 10.1136/sextrans-2013-051065

[18] Baral SD, Ketende S, Mnisi Z, Mabuza X, Grosso A, et al (2013) A cross-sectional assessment of the burden of HIV and associated individual- and structural-level characteristics among men who have sex with men in Swaziland. J Int AIDS Soc 16 Suppl 318768. 24321117 10.7448/IAS.16.4.18768 PMC3852353

[19] World Health Organization (2012) Service delivery approaches to HIV testing and counselling (HTC): A strategic HTC programme framework. Geneva, Switzerland.

[20] Maheswaran H, Thulare H, Stanistreet D, Tanser F, Newell ML (2012) Starting a home and mobile HIV testing service in a rural area of South Africa. J Acquir Immune Defic Syndr 59: e43–46. 22107821 10.1097/QAI.0b013e3182414ed7 PMC4239475

[21] Marum E, Taegtmeyer M, Parekh B, Mugo N, Lembariti S, et al (2012) “What took you so long?” The impact of PEPFAR on the expansion of HIV testing and counseling services in Africa. J AcquirImmune DeficSyndr 60 Suppl 3S63–S69. 10.1097/QAI.0b013e31825f313b 22797742

[22] Menzies N, Abang B, Wanyenze R, Nuwaha F, Mugisha B, et al (2009) The costs and effectiveness of four HIV counseling and testing strategies in Uganda. AIDS 23: 395–401. 19114865 10.1097/QAD.0b013e328321e40b

[23] Mulogo EM, Abdulaziz AS, Guerra R, Baine SO (2011) Facility and home based HIV Counseling and Testing: a comparative analysis of uptake of services by rural communities in southwestern Uganda. BMC Health Serv Res 11: 54. 21375728 10.1186/1472-6963-11-54 PMC3060861

[24] Matovu JK, Makumbi FE (2007) Expanding access to voluntary HIV counselling and testing in sub-Saharan Africa: alternative approaches for improving uptake, 2001–2007. Trop Med Int Health 12: 1315–1322. 17949401 10.1111/j.1365-3156.2007.01923.x

[25] Doherty T, Tabana H, Jackson D, Naik R, Zembe W, et al (2013) Effect of home based HIV counselling and testing intervention in rural South Africa: cluster randomised trial. BMJ 346: f3481. 23766483 10.1136/bmj.f3481 PMC3685510

[26] Naik R, Tabana H, Doherty T, Zembe W, Jackson D (2012) Client characteristics and acceptability of a home-based HIV counselling and testing intervention in rural South Africa. BMC Public Health 12: 824. 23009202 10.1186/1471-2458-12-824 PMC3487909

[27] van Schaik N, Kranzer K, Wood R, Bekker LG (2010) Earlier HIV diagnosis–are mobile services the answer? S Afr Med J 100: 671–674. 21080998 10.7196/samj.4162

[28] Nglazi MD, van Schaik N, Kranzer K, Lawn SD, Wood R, et al (2012) An incentivized HIV counseling and testing program targeting hard-to-reach unemployed men in Cape Town, South Africa. J Acquir Immune Defic Syndr 59: e28–34. 22173039 10.1097/QAI.0b013e31824445f0 PMC3801093

[29] Govindasamy D, van Schaik N, Kranzer K, Wood R, Mathews C, et al (2011) Linkage to HIV care from a mobile testing unit in South Africa by different CD4 count strata. J Acquir Immune Defic Syndr 58: 344–352. 21836524 10.1097/QAI.0b013e31822e0c4c PMC3805962

[30] Bassett IV, Govindasamy D, Erlwanger AS, Hyle EP, Kranzer K, et al (2014) Mobile HIV screening in Cape Town, South Africa: clinical impact, cost and cost-effectiveness. PLoS One 9: e85197. 24465503 10.1371/journal.pone.0085197 PMC3898963

[31] Larson BA, Schnippel K, Ndibongo B, Xulu T, Brennan A, et al (2012) Rapid point-of-care CD4 testing at mobile HIV testing sites to increase linkage to care: an evaluation of a pilot program in South Africa. J Acquir Immune Defic Syndr 61: e13–17. 22659650 10.1097/QAI.0b013e31825eec60 PMC3458178

[32] Corbett EL, Dauya E, Matambo R, Cheung YB, Makamure B, et al (2006) Uptake of workplace HIV counselling and testing: a cluster-randomised trial in Zimbabwe. PLoSMed 3: e238. 10.1371/journal.pmed.0030238 PMC1483908 16796402

[33] South African National Department of Health (2010) National HIV Counselling and Testing Policy Guidelines.

[34] Metro Trading Company (2012) Annual Report. Johannesburg, South Africa 2011/2012.

